# Nature-Based One Health Approaches to Urban Agriculture Can Deliver Food and Nutrition Security

**DOI:** 10.3389/fnut.2022.773746

**Published:** 2022-03-11

**Authors:** Bassey Ebenso, Akaninyene Otu, Alessandro Giusti, Philipe Cousin, Victor Adetimirin, Hary Razafindralambo, Emmanuel Effa, Vasileios Gkisakis, Ousmane Thiare, Vincent Levavasseur, Sonagnon Kouhounde, Kifouli Adeoti, Abdur Rahim, Majid Mounir

**Affiliations:** ^1^Leeds Institute of Health Sciences, University of Leeds, Leeds, United Kingdom; ^2^Foundation for Healthcare Innovation and Development (FHIND), Calabar, Nigeria; ^3^Department of Internal Medicine, University of Calabar, Calabar, Nigeria; ^4^Hull University Teaching Hospital, Hull, United Kingdom; ^5^CyRIC-Cyprus Research and Innovation Center Ltd., Nicosia, Cyprus; ^6^SENSEEN, Nice, France; ^7^Department of Crop and Horticultural Sciences, University of Ibadan, Ibadan, Nigeria; ^8^ProBioLab, Gembloux Agro-Bio Tech, University of Liège, Liège, Belgium; ^9^Institute of Olive Tree, Subtropical Crops & Viticulture, Department of Olive and Horticultural crops, ELGO – DIMITRA, Kalamata, Greece; ^10^Université Gaston Berger de Saint Louis, Saint-Louis, Senegal; ^11^CAN (National Centre Agroecology) Ver deTerre, Paris, France; ^12^Laboratory of Applied Biologic Sciences, Université Aube Nouvelle, Bobo-Dioulasso, Burkina Faso; ^13^Laboratoire de Microbiologie et de Technologie Alimentaire (LAMITA), Faculté des Sciences et Techniques, Université d’Abomey-Calavi, Cotonou, Benin; ^14^WAZIUP, Trento, Italy; ^15^Department of Food Science and Nutrition, Biotransformations Laboratory, Hassan II Institute of Agronomy and Veterinary Medicine, Rabat Instituts, Rabat, Morocco

**Keywords:** nature-based, one health, urban agriculture, food and nutrition security, climate-smart, postbiotic

## Abstract

The increasing global human population is projected to reach 9.7 billion people by 2050. This population growth is currently linked to the trends of world-wide urbanization, growth of megacities and shifting dietary patterns. While humankind faces the daunting challenge of feeding and providing healthy lives for its teeming populations, urban agriculture holds promise for improving the quality of life in cities. Fortunately, policymakers and planners are accepting the need to support peri-urban farmers to increase the resilience of food systems while efficiently managing already strained natural resources. We argue that for urban agriculture to significantly increase food yields, it is crucial to adopt a One Health approach to agriculture and environmental stewardship. Here, we propose six nature-based and climate-smart approaches to accelerate the transition toward more sustainable food systems. These approaches include reducing the reliance on synthetic agricultural inputs, increasing biodiversity through producing locally adapted crops and livestock breeds, using probiotics and postbiotics, and adopting portable digital decision-support systems. Such radical approaches to transforming food production will require cross-sectoral stakeholder engagement at international, national, and community levels to protect biodiversity and the environment whilst ensuring sustainable and nutritious diets that are culturally acceptable, accessible, and affordable for all.

## Introduction

Food insecurity was on the rise before the COVID-19 pandemic, with an estimated 25.9% of the global population (about 2 billion people) affected by moderate or severe food insecurity in 2019, an increase from 22.4% in 2014 (1). The COVID-19 pandemic has worsened household production and access to safe and nutritious food, thus threatening food systems already impacted by pre-existing or seasonal threats and vulnerabilities such as natural hazards, pests, armed conflicts and violence (2). Additionally, climate change has precipitated extreme weather conditions characterized by extended periods of drought that are rapidly succeeded by heavy rains and storms. These extreme weather events have ravaged crop production for consecutive years, leaving small-scale farmers without food for their families or goods to sell. Evidence shows that climate change is reducing food supplies when crop yields are converted into consumable calories on people’s plates (3). For instance, annual food calories have declined by between 0.8 and 2.2% annually in Asian countries such as India and Nepal. Similarly, losses are occurring in African countries such as South Africa (∼8%), Ghana (∼8%), and Zimbabwe (∼10%) as well as in European countries such as Italy (∼7%), France (∼7%), Germany (∼11%) and Ireland (∼12%) (4). It is clear that judgment of quality varies with setting or context and calorie intake is not the only criterion to consider. However, although food needs to provide nutrients, be safe and widely acceptable, caloric intake remains a key criterion in the context of food insecurity (5, 6).

Current global estimates also indicate that 820 million people are still hungry (7) and at least 2 billion more lack sufficient micro-nutrients (8). The most affected by hunger are often women and those employed in informal economies. The foregoing highlights the significance of SDG-13 (climate action) for attaining SDG-2 (End hunger, achieve food security and improve nutrition and promote sustainable agriculture), as well as the importance of SDG-2 for attaining SDG-3 (Ensure healthy lives and promote well-being for all at all ages).

A 2012 report of the United Nations Committee on World Food Security (9) stipulated the yardstick for food security as: “*when a person has secure access to food which is safe and consumed in sufficient quantity and quality to meet his/her dietary needs and food preferences, in a hygienic environment supported by adequate healthcare delivery to ensure he/she enjoys a healthy and active life free from malnutrition”* (10). This yardstick identifies three main determinants of nutrition security for individuals as: access to food, care and utilization, and health and sanitation. This implies that the complex interplay of factors at sub-national, national, and global levels can influence the access to food, health services and good hygienic conditions in communities and households.

### Existing Agricultural Practices Threaten Global Food Security

Past efforts at addressing world hunger and malnutrition have focused on producing more food at an industrial scale. Yet, extractive intensification of food production has failed to adequately feed the world. Instead, industrial agriculture, which ignores linkages to localized food systems, has had negative impacts on wetlands and wildlife habitats (11). This has given rise to pesticide-resistant pests whilst increasing the risk of disease spread. Agricultural intensification has relied on increasing amounts of chemical inputs to boost unsustainable yields, killing soils and drastically reducing the diversity of both plant and animal species with deleterious effects on human health (12, 13).

The adverse effects have been linked to activities such as the addition of lime which alters the soil pH and disrupts the biota. Also, the bacterial: fungal ratio is affected by the addition of fertilizers and manures which alter the C:N ratio, and the act of tilling soils breaks down the fungal hyphens with far reaching effects. Conversely, some animal production systems can be anthropogenic drivers of climate change, water pollution and biodiversity losses (14). Indeed, these intensive mono-species production systems have contributed to a decline in agrobiodiversity and reduced their capacity for resilience in the face of global changes (15).

### Role of Peri-Urban Farming in Global Food Production

Feeding the world’s population (7.7 billion people) sustainably will require considerable increases in urban and peri-urban food production (16). The quest to feed the world’s growing population is triggering worldwide migration of rural people to cities. Today, 55% of the planet’s inhabitants live in cities, and this proportion is projected to increase to 68% by 2050 (16, 17), with 80% of all food produced globally destined for consumption in urban spaces. Rapid migration of farmers and unemployed youths from isolated rural and peri-urban areas to Metropolitan cities has generated high-density slums in the periphery of cities (18) characterized by poor housing, lack of clean water and poor hygiene and sanitation facilities that promote the spread of emerging infectious diseases such as cholera, salmonellosis, SARS and Ebola (19, 20).

For this manuscript, urban agriculture denotes the integration of cultivation, processing and distribution of food into the urban economy and ecological system (21), for the purpose of providing fresh food, generating employment, recycling waste and strengthening cities’ resilience to climate change (22). Urban agriculture therefore holds promise for minimizing some of the above negative impacts and improving the quality of life of urban inhabitants. However, the contribution of urban agriculture to feeding the teeming populations of cities (23) is often overlooked in the context of complex global supply chains that enable some people to obtain food from anywhere in the world.

Fortunately, policymakers and planners are beginning to accept the need to support peri-urban farmers to strengthen the resilience of food systems while efficiently managing already strained natural resources and absorbing the pressures on infrastructure (24). Although cities account for only 2–3% of the earth’s surface, they utilize about 78% of the world’s energy and produce over 60% of greenhouse gas emissions (25). Cities also consume huge quantities of water, pollute the environment, and generate excessive amounts of waste products (26).

## Transforming Urban Food Systems Through Sustainable One Health Approaches

Against the backdrop of population growth, the interconnectedness of agricultural practices and the health of plants, animals and people call for a nature-based, One Health approach (27, 28) to ending hunger, achieving food and nutrition security and promoting sustainable agriculture. The One Health approach to food production will involve working with stakeholders across academic, government and private institutions to transform global food systems. This approach supports the Food and Agricultural Organization’s framework for the Urban Food Agenda 2030 that promotes a multi-sectorial, multi-stakeholder and multi-level approach to food insecurity and malnutrition across the rural-urban continuum (22). Below, we outline climate-smart approaches with some examples for strengthening food systems:

### Ecological Practices That Optimize Limited Urban Spaces and Minimize Synthetic Inputs

Agroecology is based on participatory interactions between traditional knowledge and modern science. It provides a a path for the radical redesign and reconstruction of the dominant monoculture production systems into resilient farming systems, capable of coping with widespread disruptions (29). The challenges of the lack of urban land space for cultivation, and long distances of agricultural sectors from urban centers provide opportunities for developing urban agriculture. Urban farmers have adopted different soilless techniques to complement conventional growing techniques in soil.

Similarly, land-related constraints have been overcome through growing plants without soil, using inert media such as coconut fiber, clay pebbles or with any media (such as water and soluble nutrients) (23). For example, farmers in Paris, France have started the world’s largest soil-free rooftop farm (30), cultivating: (i) strawberries in vertical plastic columns without soil, and (ii) lettuces, tomatoes, aubergines and herbs in horizontal trays packed with coconut fiber. The network “Maraîchage Sol Vivant” (Living Soil Vegetable Gardening) in France has demonstrated the possibility of feeding 100 persons with fresh vegetables grown on 5000m^2^. Therefore agroecological farming on 10% of the arable area of the Paris region could provide enough to feed this megalopolis. Such efforts could be complemented by changes in dietary habits with a significant reduction in the consumption of animal products, particularly in climes where there is an overdependence on livestock-derived foods. These dietary shifts are not appropriate for low- and middle-income countries where many people, and especially the poorest, consume none or very small amount of livestock-derived foods. Another example of nature-based and sustainable model of agriculture is found in in Tel Aviv, Israel where “Green in the City” a hydroponics and aquaponics (31) company has integrated the concept of vertical farming into urban food systems by providing farmers with space on high-rise buildings to grow vegetables in floating beds of water without pesticides. Such practices can ultimately reduce other greenhouse gases – GHG (NO_2_, CH_4_), yield improvement, reduce fertilizer required, reduce fuel consumption and improve urban water quality (32). While hydroponic systems are essential today to maximize production and increase yields, less information is available on the impact of hydroponic methods on the nutritional status of productions and in particular on their levels of bioactive compounds (33).

### Increasing Crop Diversity Through Farming Locally Adapted Crops

Biodiversity, is a central principle of agroecology for increasing/maintaining productivity and resilience while encouraging optimal levels of wildlife for ensuring basic natural processes. Two popular methods of biodiversity include: (i) crop diversity approaches involving simultaneous cultivation of two or more grains and/or vegetables on the same field to increase productivity on a farmland by making use of inputs that would otherwise be utilized by a single crop; (ii) crop rotation approaches that help to increase temporal diversity. Other methods of biodiversity include enhancing crop genetic diversity, and diversifying landscapes (for example, by planting trees to increase carbon sequestration) surrounding croplands.

There is consistently strong evidence (34) that strategically increasing plant diversity increases crop and forage yield, wood production, yield stability, pollinators, weed suppression, and pest suppression. An example of successful application of crop genetic diversity to enhance yield stability in a tropical context is seen in Nigeria where the recent development of super-sweet maize hybrids (35) as well as provitamin A quality protein maize (36) have potential to diversify the maize value chain. This chain was previously dominated by flint and dent endosperm types and this offers some diversity in nutrition and business opportunities. Additionally, the super-sweet hybrids are showing signs of better adaptation and resistance to local diseases compared to plants raised from seeds of imported varieties. These locally developed, first generation super-sweet maize hybrids are currently undergoing multi-site trials in Nigeria. As well as being environmentally friendly, the cultivation of resistant varieties is cheaper as they add little or nothing to production beyond the cost of seeds (37). Similarly, cassava varieties (TMS 98/0583 and TMS 98/0505) have been developed in Nigeria to resist cassava mosaic virus and enhance yield stability. These environmentally friendly cassava varieties are generally cheaper and longer-lasting with potential to reduce post-harvest losses of agricultural products and food waste that commonly occurs in developing countries.

Prime crop candidates for urban farming in non-tropical contexts include rice, tomatoes, peppers, millet, spinach, radishes, wheat, oat, barley and turnips. An example of successful application of increasing crop diversity is the work of Jean–Martin Fortier in Canada which involves the intensification of crop rotation to generate more income and less weed management (38). Agroforestery, and in particular peri-urban forest gardens, is another crop intensification strategy which involves mixing crop stages with numerous other ecosystemic services.

### Using Probiotics and Postbiotics to Manage Viral Diseases in Animals and Humans

**FIGURE 1:**
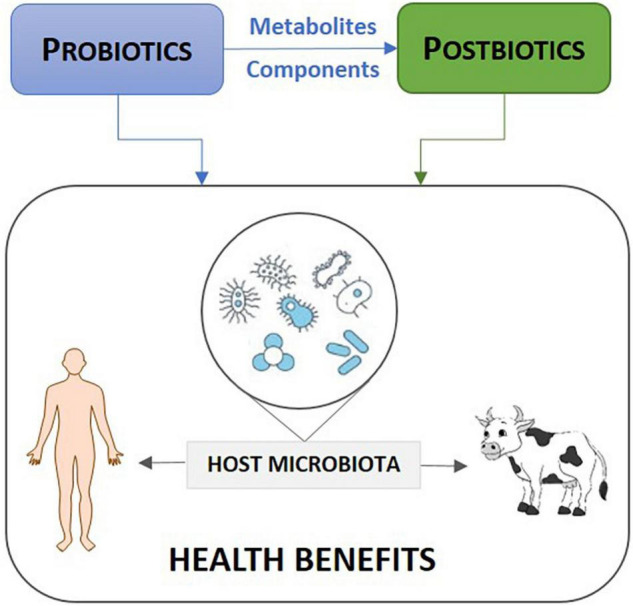
Use of probiotics and postbiotics to manage animal and human disease.

Peri-urban areas are highly vulnerable to emerging infectious disease, owing to fast growing environments and high climate change sensitivity. Moreover, livestock production systems of indigenous and non-indigenous breed types, including ruminants and monogastric animals, are often diverse and dynamic in these spaces, but typically under-governed. As a consequence, peri-urban areas easily undergo important environmental change while being highly susceptible to diverse zoonotic diseases (39). The use of probiotics and postbiotics for controlling enteric and respiratory viral infections has gained huge attention recently (40). Whereas probiotics are live microbial cells generated from food and non-food sources (41), postbiotics are inanimate microbial cells, cell components, and/or their metabolites (see Figure 1), used to confer health benefits on animals and humans (42). The antiviral activities of probiotics and postbiotics is linked to their interaction with viruses and the production of inhibitory substances such as bacteriocins, enzymes and peptides or the stimulation of host immune system (43).

For nutritional purposes or medical foods, probiotics are Generally Recognized as Safe (GRAS) and have the status of Qualified Presumption of Safety (QPS) when the strain belongs to a widely characterized species in terms of safe usage (44). The administration of postbiotics offers natural, simple, cost-effective, and safe solutions for controlling and/or lowering risks of disease outbreaks (45, 46). The use of probiotics to transform food systems is seen in East Africa where women dairy farmers have been empowered to produce probiotic fermented food as a pathway to generating income for financial independence (SDG-1), and providing nutritious food for families and communities (SDG-2) (47). By increasing access to probiotics, this nature-based initiative for sustainable agriculture led to 262 production units reaching 260,000 weekly consumers in Uganda, Kenya and Tanzania.

### Using Locally Adapted Livestock Breeds in the Urban Environment

Livestock can positively or negatively impact the ecosystem, depending on the quality of management practices adopted by urban farmers. Four successful practices adopted by farmer networks in France to increase the use of locally adapted livestock breeds include: (i) adapting feed rations to reduce external inputs and get healthier animals that yield healthier products such as milk, eggs or meat richer in omega 3 and unsaturated fat; (ii) using non-oxidant alimentation to regulate animal and human health, (iii) developing rotational grazing to produce more forage with higher quality, (iv) recycling urban waste and sludge to get organic fertilizer. However, quality assurance will be required to confirm the suitability of recycled urban waste and sludge for organic production.

On the other hand, livestock adaptation practices in Nigeria have successfully integrated poultry or pig keeping with fish and rice cultivation to reduce, reuse and recycle waste. Waste products from one component (e.g., poultry) is used as an input for other components (fish and rice production). Such practices led to improved adaptation of local breeds of poultry (the FUNAAB Alpha breed) and fish species such as the African catfish (*Clarias gariepinus*) and the Nile tilapia (*Oreochromis nitilocus*). Nonetheless, care is required to minimize the transmission of zoonotic diseases such as salmonellosis, Campylobacteriosis and highly pathogenic avian influenza. The use of farm waste mixed with synbiotics as functional feeds can promote animal health. Similarly, using manure as fertilizer on grasslands and croplands can limit external inputs and combat medium and long-term effects on climate, environment, and animal welfare. It is crucial to monitor results of livestock adaptation practices by collecting field data to track improvement in pedoclimatic zones (48).

### Enhancing Diversity Within Animal Production Systems to Strengthen Resilience

Diversity within animal production systems can be improved through two major axes. First, through diversifying components of the system such as multispecies grasslands and animal inter-individual variability. Second, through improving interactions between components by integrating plant and animal production and the multi-species animal production systems (14). Efficient management of different combinations of these components will increase resilience of animal production systems.

For example, rearing different animal species such as cattle and sheep offers a risk-spreading strategy against droughts, disease outbreaks and market price fluctuations. Adapting management practices to the biological characteristics of each species facilitates resilience by modulating breeding practices based on climate sensitivity and female longevity. A diversity of forage resources also helps secure the feeding system against seasonal and long-term climatic variability. In agro-pastoral systems commonly practiced in peri-urban environment, the feeding system is based on complementarities between cultivated grasslands (which are used to secure animal performance in crucial periods such as mating or lactation) and rangelands (which are mostly grazed at times when the animals have low nutrient requirements) (49). When the availability of feed resources is limited or unpredictable, defining seasonal priorities between animals with high requirements (which will need to be given priority access to the best resources), and animals with low requirements, is a helpful approach to designing efficient feeding systems.

### Adopting Digital Technology to Support Agroecological Principles and Strategies

Increasing acceptance of Urban Agriculture (UA) by donors, researchers and development organizations is stimulated technical experimentation and use of information and communication technologies (ICTs) to accelerate transition toward sustainability in food systems (50). ICTs are shaping the organization, integration, coordination and the increasing transparency of food chains at global, regional, and local levels thereby reducing transaction costs the food chain (51). However, ICTs appear to be used differently in LMICs compared to in Europe and the Americas. For example, ICTs are mainly used in Africa to access and disseminate livestock husbandry information and to improve livestock husbandry practices among peri-urban farmers (52). Different types of low-cost ICTs such as mobile phones, the internet and social media platforms are used in Tanzania to empower peri-urban farmers to access agricultural information. Within such urban contexts, ICTs enhance community engagement through promoting connections between peer food networks and influence consumption and production practices, preferences, habits and decisions (52). In this sense, these social networks of urban and peri-urban farmers facilitate and accelerate the democratization of information sharing by using horizontal channels that bypass rigid institutional norms (50). Moreover, ICTs can bring producers and customers closer to urban food systems to increase collaboration, promote market adjustments, and lower carbon footprint of food production (53).

**FIGURE 2:**
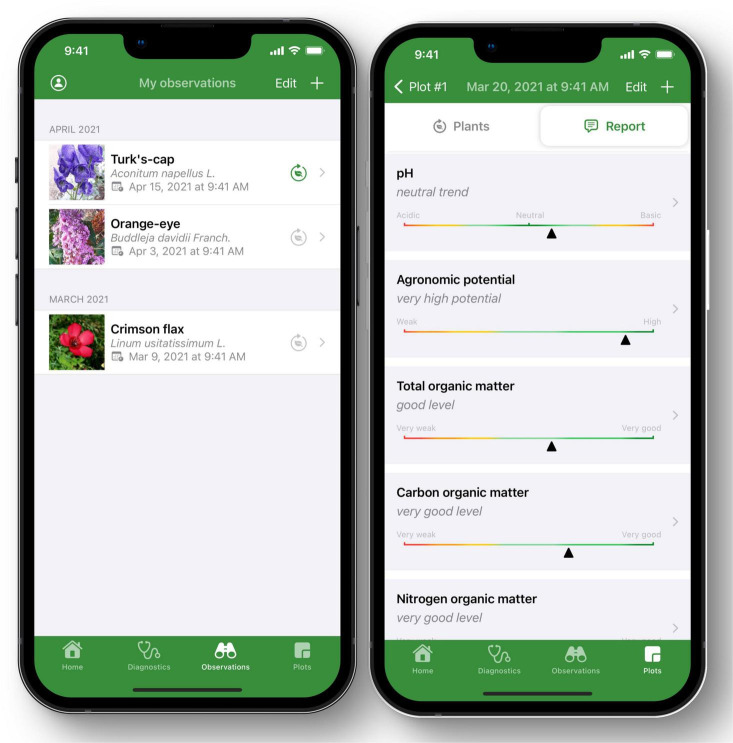
Output from the AGRODIAG mobile artificial intelligence (IA) app showing for instance total organic matter, agronomic potential, pH to give soil assessment for taking measures. Photo Credit: Ver de Terre Digital.

User-friendly and reliable measurement solutions (54), such as Decision Support Systems (DSS) and other innovative digital technology can support transition to agroecology. This can be done either directly (for example, robotic weeding without using chemicals) or indirectly (for example, supporting optimal application of bio-pesticides and bio-fertilizers based on precise but fast measurements). The DSS have the potential to facilitate: (a) farmers’ decision-making about adopting agroecology, (b) impact assessment of the applied methods, (c) standardization and regulation of efficient approaches, (d) communication between farmers and other actors, and (e) track market trends. Data-based decision-support mechanisms that support agroecology are largely unavailable at present, although some European initiatives are trying to bridge this gap. For instance, using a database of over 30,000 plant images and botanist Gérard Ducerf’s analysis method (55), a mobile artificial intelligence (IA) app has been developed called AGRODIAG (see soildiag on Google store) to provide a soil diagnostics from the analysis of the bio-indicator plants found in a particular spot (Figure 2). The app provides detailed reports with key indicators like organic matter, pollution, redox, biological activity and more. Each diagnostic is time stamped and geolocalized. Users have a complete history of all the observations and diagnostics they have made and can organize them by plots.

This use case shows the use of DSS with big data, expertise and AI systems to recognize plants and soil diagnostics. It provides evidence of the potential of digitalization and the benefits that can be achieved using simple mobile phones.

**FIGURE 3:**
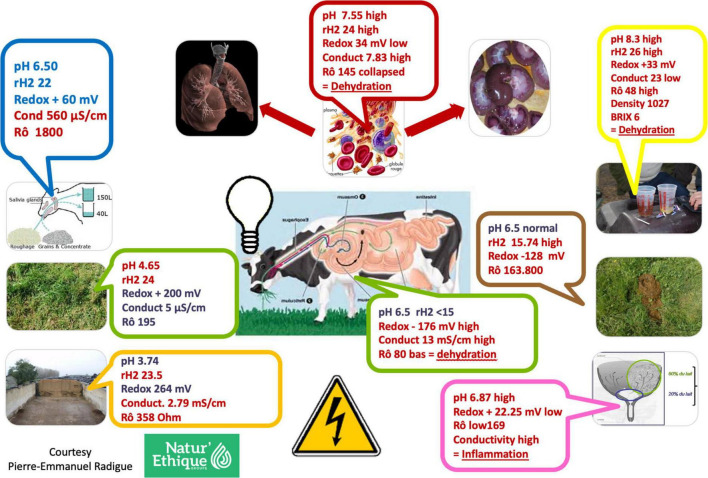
Example of disorders analyzed by Eh-pH on soil, plant, and livestock.

**FIGURE 4:**
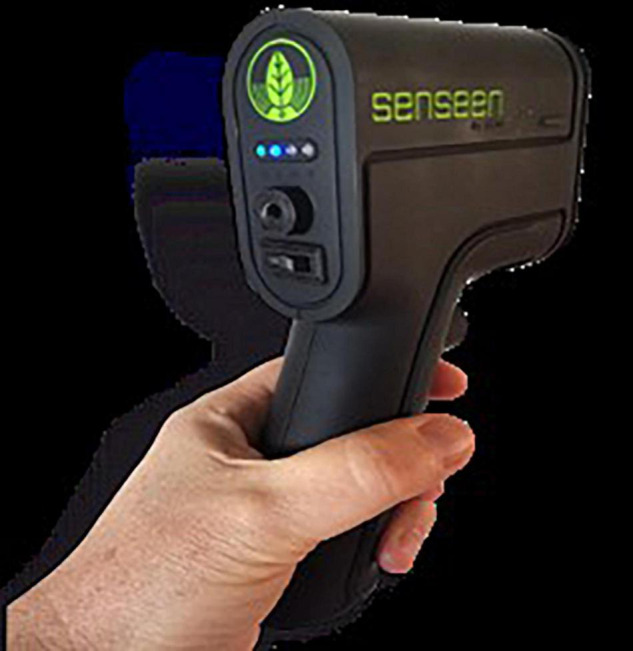
Low-cost NIR spectrometer prototype for measuring Eh-pH.

Also new portable technologies (see Figure 3) can also help to side-step the over-reliance on laboratory measurement of indicators for understanding new agroecological environments linked to soils and livestock production. New indicators such as redox potential or Eh (assessing the availability of electrons) and pH (assessing the availability of protons) can be identified using sensors or Internet of Things (IoT) to obtain data for analytics and indicators not yet measured (Figure 4). As soil health or quality is related to soil organic matter, nutrient cycle, biological activity and soil structure, these parameters can be measured by tracking soil redox potential and pH levels (56, 57). These same parameters (Eh and pH) are related to, and can explain fundamental processes not only in soils and plants, but in animals and humans too. Thus, we proposed to use Eh-pH as indicators for monitoring plant health and that of livestock (shown center of Figure 4). Various methods developed to assess plant health such as chlorophyll fluorescence, photo-oxidative stress markers (including photosynthetic pigments, photosystem II efficiency, reactive carbonyl species, and antioxidant systems) are all related to Eh and pH (58). By Using Eh-pH levels as indicators both for plant and animal health which is easily measured, (e.g., one-click measurement with a portable low cost device), the approach has potential for enabling the adoption of a One Health approach to urban agriculture (59). However, electrochemistry-based measurements of these parameters can be limited by imprecisions in measurements. There is an urgent need, therefore, to develop portable low-cost, near infra-red (NIR) tools for *in situ* rapid and reliable measurement of Eh and pH. The availability of unique portable devices which measure Eh-pH easily (using spectrometry) has facilitated the understanding and control of the environment.

## Discussion and Recommendations

Efforts to institutionalize One Health initiatives have been supported by global institutions including the World Health Organization, the African Union, the European Union (with the One Health European Joint Programme), and the United Nations Food and Agricultural Organization. However, the effectiveness of the nature-based, and climate smart One Health approaches proposed in this manuscript will depend on coordinated and inclusive multi-stakeholder processes involving ministries of health, agriculture, environment, forestry, urban planning etc.). It will be vital to foster public–private partnerships which promote resource sharing, and economies of scale for One Health to reduce the financial burden on national governments. Country-level policies on food and nutrition security which also includes food safety, should reflect the principles of agroecology and environmental stewardship and embedded into One Health training curricula and implementation plans.

**FIGURE 5:**
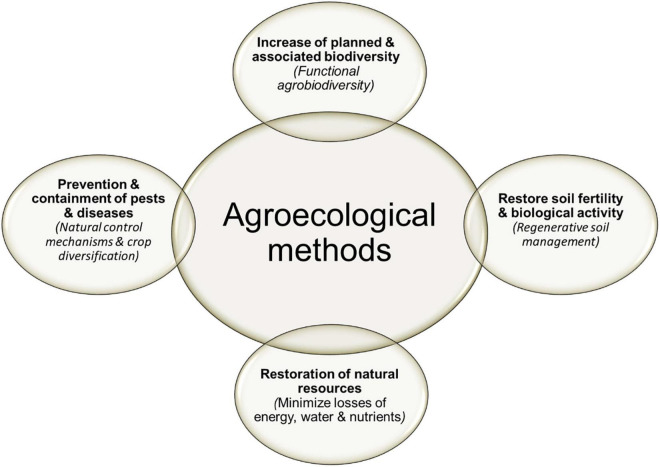
Agroecological methods generating ecosystems services related to health of ecosystems, animals, and people.

Basic agroecological strategies are required to increase health of ecosystems, animals and people [see Figure 5; adapted from Altieri (29); Bàrberi (60)]. This should involve:

(i) Increase of spatial and temporal “planned” (i.e., proper diversification of cultivated crops) and “associated” biodiversity (i.e., enhancement and introduction of beneficial wild flora and fauna) at gene, species and landscape level, collectively referred as “functional agrobiodiversity”;
(ii) Prevention and containment of pests and diseases through natural control mechanisms and crop diversification practices;
(iii) Regenerative soil management to restore soil fertility and biological activity;
(iv) Restoration of natural resources to minimize losses of energy, water and nutrients.

## Conclusion

Identification and production of stress-tolerant crops and livestock at sub-national and community levels will promote diversification of urban food systems and ultimately strengthen the resilience of agroecosystems. This could potentially mitigate the deleterious effects of climate change (SDG-13) and promote food and nutrition security for households (SDG-2), and good health for all (SDG-3). One Health approaches to urban and peri-urban agriculture have a vital role to play but it cannot deliver food and nutrition security alone – culturally appropriate, context-specific and feasible shifts in dietary patterns are required so that there is neither over- nor under-consumption of livestock foods. The necessary interplay between urban/peri-urban and rural agriculture will need to be managed by promoting collaborative approaches involving cross-sectoral agencies, civil society organizations, farmers and national centres for disease control (CDC). Only then will we successfully deliver food and nutrition security on a global scale.

## Data Availability Statement

The original contributions presented in the study are included in the article/supplementary material, further inquiries can be directed to the corresponding author.

## Author Contributions

BE and AO conceptualized the article and provided the outline for the article. All authors contributed to various sections of the article and read and approved the final draft.

## Conflict of Interest

PC was employed by the company SENSEEN. AR was employed by the company WAZIUP. AG was employed by the company CyRIC-Cyprus Research and Innovation Center Ltd., Nicosia, Cyprus. The remaining authors declare that the research was conducted in the absence of any commercial or financial relationships that could be construed as a potential conflict of interest.

## Publisher’s Note

All claims expressed in this article are solely those of the authors and do not necessarily represent those of their affiliated organizations, or those of the publisher, the editors and the reviewers. Any product that may be evaluated in this article, or claim that may be made by its manufacturer, is not guaranteed or endorsed by the publisher.
